# Intense simplified strategy for newly diagnosed type 2 diabetes in patients with severe hyperglycaemia: multicentre, open label, randomised trial

**DOI:** 10.1136/bmj-2024-080122

**Published:** 2024-10-15

**Authors:** Liehua Liu, Weijian Ke, Hai Li, Fangping Li, Guanjie Fan, Jian Kuang, Jianhua Ma, Xiuwei Zhang, Bing Ji, Shu Li, Yinghong Du, Yaoming Xue, Zhaohui Lyu, Leili Gao, Shen Qu, Yongquan Shi, Li Yan, Wanping Deng, Chaoyan Xu, Peiji Dai, Lijuan Xu, Juan Liu, Xuesi Wan, Guohong Wei, Shuang Yu, Shubin Hong, Pengyuan Zhang, Zhimin Huang, Xiaopei Cao, Zhihong Liao, Haipeng Xiao, Yiming Mu, Yehuda Handelsman, Yanbing Li

**Affiliations:** 1 The First Affiliated Hospital of Sun Yat-sen University, Guangzhou, Guangdong Province, China; 2 The Seventh Affiliated Hospital of Sun Yat-sen University, Yantian District, Shenzen, Guangdong Province, China; 3 Guangdong Provincial Hospital of Traditional Chinese Medicine, Guangzhou, Guangdong Province, China; 4 Department of Endocrinology, Guangdong Provincial People's Hospital (Guangdong Academy of Medical Sciences), Southern Medical University, Guangzhou, Guangdong Province, China; 5 Nanjing First Hospital, Nanjing, Jiangsu Province, China; 6 The Tenth Affiliated Hospital, Southern Medical University (Dongguan People’s Hospital), Wanjiang District, Dongguan City, Guangdong Province, China; 7 Clifford Hospital, Panyu District, Guangzhou, Guangdong Province, China; 8 Huizhou Municipal Central Hospital, Huizhou, Guangdong Province, China; 9 The Affiliated Panyu Central Hospital of Guangzhou Medical University, Panyu district, Guangzhou, Guangdong Province, China; 10 Southern Medical University Nanfang Hospital, Guangzhou, Guangdong Province, China; 11 Department of Endocrinology, The First Medical Center, Chinese People’s Liberation Army General Hospital, Haidian District, Beijing, China; 12 Peking University People's Hospital, Xicheng District, Beijing, China; 13 Shanghai Tenth People's Hospital of TongJi University, Shanghai, China; 14 Shanghai Changzheng Hospital, Shanghai, China; 15 Sun Yat-sen Memorial Hospital of Sun Yat-sen University, Guangzhou, Guangdong Province, China; 16 Metabolic Institute of America, Tarzana, CA, USA

## Abstract

**Objective:**

To evaluate whether the intense simplified strategy, which comprises short term intensive insulin therapy (SIIT) followed by subsequent oral antihyperglycaemic regimens, could improve long term glycaemic outcomes in patients with newly diagnosed type 2 diabetes mellitus and severe hyperglycaemia.

**Design:**

Multicentre, open label, randomised trial.

**Setting:**

15 hospitals in China between December 2017 and December 2020.

**Participants:**

412 patients with newly diagnosed type 2 diabetes and significant hyperglycaemia (HbA_1c_ ≥8.5%).

**Interventions:**

All randomised participants initially received SIIT for 2-3 weeks, followed by linagliptin 5 mg/day, metformin 1000 mg/day, combination linagliptin plus metformin, or lifestyle modification alone (control) for 48 weeks.

**Main outcome measures:**

The primary outcome was the percentage of participants achieving HbA_1c_ <7.0% at week 48 after SIIT. Secondary outcomes included glycaemic control, β cell function, and variations in insulin sensitivity.

**Results:**

412 participants were randomised. At baseline, the mean age was 46.8 (standard deviation 11.2) years, mean body mass index was 25.8 (2.9), and mean HbA_1c_ was 11.0% (1.9%). At week 48, 80% (78/97), 72% (63/88), and 73% (69/95) of patients in the linagliptin plus metformin, linagliptin, and metformin groups, respectively, achieved HbA_1c_ <7.0%, compared with 60% (56/93) in the control group (P=0.02 overall; P=0.003 for linagliptin plus metformin versus control; P=0.12 for linagliptin versus control; P=0.09 for metformin versus control). Additionally, 70% (68/97), 68% (60/88), and 68% (65/95) of patients in the linagliptin plus metformin, linagliptin, and metformin group, respectively, achieved HbA_1c_ <6.5% compared with 48% (45/93) in the control group (P=0.005 overall; P=0.005 for linagliptin plus metformin versus control; P=0.01 for linagliptin versus control; P=0.008 for metformin versus control; all were significant after adjustment for multiple comparisons). Thus, compared with the control group, participants in the linagliptin plus metformin group were more likely to achieve HbA_1c_ <7.0% at week 48 (odds ratio 2.78, 95% confidence interval 1.37 to 5.65; P=0.005). Moreover, the linagliptin plus metformin group showed the most significant improvement in fasting plasma glucose and β cell function indices. All treatments were well tolerated.

**Conclusions:**

The intense simplified strategy using subsequent oral therapies post-SIIT, especially the linagliptin plus metformin combination, sustainably improved glycaemic control and β cell function in patients with newly diagnosed type 2 diabetes mellitus and severe hyperglycaemia. This approach offers a promising direction for decision making in the clinical management of type 2 diabetes mellitus.

**Trial registration:**

ClinicalTrials.gov NCT03194945

**Figure:**
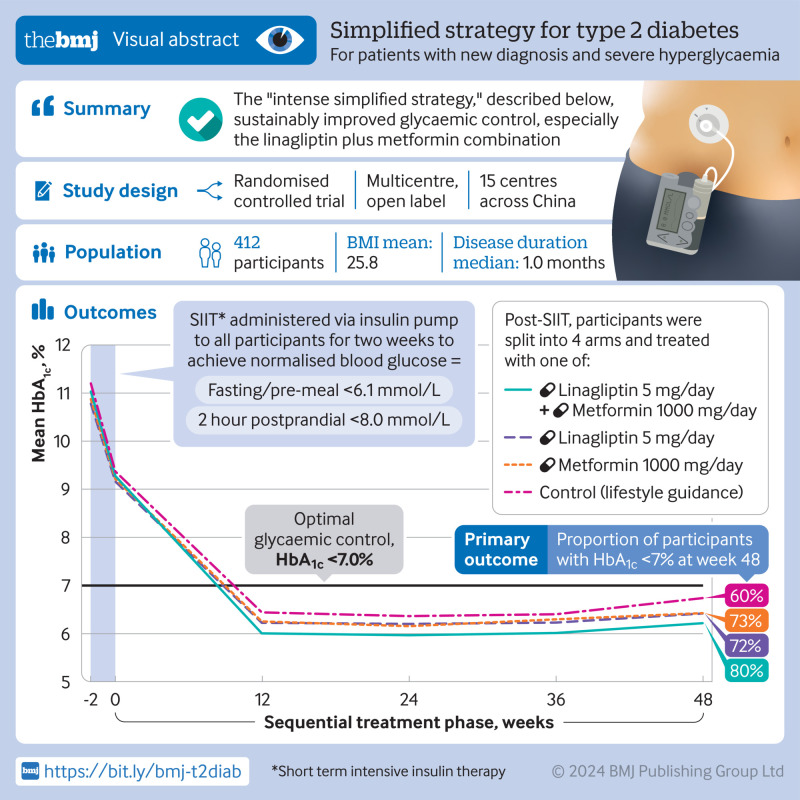


## Introduction

As estimated by the International Diabetes Federation, the global prevalence of diabetes has reached 537 million.^1^ Exposure to hyperglycaemia, even in the first year after diagnosis, is a primary risk factor for chronic complications of diabetes and even death. These effects can persist for decades and are known as the legacy effect.^2^ ^3^ ^4^ As discovered recently by the 24 year follow-up of the UK Prospective Diabetes Study, early intensive glycaemic control provides a near lifelong reduction in the risk of death and of microvascular and macrovascular complications.^5^ However, despite various glucose lowering treatments, more than half of the patients had inadequate glycaemic control,^6^ indicating that traditional treatment strategies that escalate treatment intensity in a stepwise manner to overcome deteriorating hyperglycaemia are unable to delay β cell failure and progression of diabetes.

Significant hyperglycaemia is common in patients with newly diagnosed type 2 diabetes mellitus. Owing to the limited efficacy of monotherapy, the American Diabetes Association and the European Association for the Study of Diabetes recommend that combination therapy should be used when the glycated haemoglobin A_1c_ (HbA_1c_) levels exceed treatment targets by 1.5% (≥8.5% for most patients), with insulin therapy needed if HbA_1c_ exceeds 10% (evidence level E; expert consensus or clinical experience).^7^ Nevertheless, standardised treatment pathways for these patients remain unclear.

Emerging evidence has shown that the progression of type 2 diabetes is not irreversible, especially in the early stages.^8^ ^9^ Short term intensive insulin therapy (SIIT) has shown immediate and substantial benefits in early type 2 diabetes. Two to three weeks of SIIT significantly improves β cell function and insulin sensitivity by eliminating glucotoxicity, inducing remission of diabetes for longer than a year in more than 50% of patients with newly diagnosed type 2 diabetes.^10^ ^11^ ^12^ ^13^ In most studies of SIIT, benefits were observed in patients with significant hyperglycaemia (mean HbA_1c_ ≥10%) and mild overweight (mean body mass index approximately 25).^10^ ^12^ ^13^ ^14^ Nevertheless, the remission rate declined over time, from 70% immediately after SIIT to roughly 50% at the end of the first year and around 40% at the end of the second year.^15^ This provides a rationale for investigating whether simplified oral regimens subsequent to SIIT could help to maintain the good glycaemic control induced by SIIT by persistently avoiding the detrimental effects of glucotoxicity and circumventing the disadvantages of intricate glucose lowering regimens.

In this context, we proposed an intense simplified strategy, which uses SIIT as the initial therapy followed by simplified oral anti-diabetes regimens as maintenance therapy. We hypothesised that this strategy could improve glycaemic control and preserve β cell function in patients with newly diagnosed type 2 diabetes and severe hyperglycaemia. We thus conducted this nationwide, multicentre, randomised controlled trial to evaluate the effect of different subsequent regimens (metformin, linagliptin, or their combination) after SIIT on glycaemic outcomes over 48 weeks, with lifestyle modification alone as the control.

## Methods

### Study design and participants

This is an open label, nationwide, multicentre, randomised controlled trial conducted across 15 centres in China. The participants, whose diagnosis of type 2 diabetes was newly made according to the World Health Organization’s criteria in the research centres, had to be aged between 20 and 70 years, be naïve to antihyperglycaemic drugs and had not previously received any systematic diabetes related medical advice and interventions, had body mass index of 22.0-35.0, had fasting plasma glucose between 7.0 mmol/L and 16.7 mmol/L, and had HbA_1c_ ≥8.5% at screening. Disease duration was estimated on the basis of the onset of clinical symptoms or the period of elevated blood glucose reported by the participants. Major exclusion criteria included the presence of glutamic acid decarboxylase antibodies, use of drugs that affect glucose metabolism, acute complications of diabetes (for example, diabetic ketoacidosis or hyperosmolar hyperglycaemic state), diabetic proliferative retinopathy, albumin excretion >300 mg/day or cardiovascular events within the preceding six months, alanine transferase ≥2.5 times the upper limit of normal, an estimated glomerular filtration rate of ≤60 mL/min, or any significant systemic diseases as determined by physical examination or medical history. The trial followed the principles of the Declaration of Helsinki and Good Clinical Practice guidelines of the International Conference for Harmonization. All participants provided signed informed consent.

### Randomisation and masking

After enrolment, eligible patients were randomised 1:1:1:1 into four subsequent treatment arms, stratified by the participating centres. A statistician not involved in the study encoded and prepared random envelopes, which were then distributed to each centre and stored by the file administrator. After obtaining informed consent from the participant, the file administrator retrieved and opened the envelope in the presence of the investigators. Afterwards, the assignment was verified and archived in the participant's file for future reference. Both the researchers and patients were unaware of the allocation before randomisation but were informed afterwards.

### Procedures

At baseline, we assessed anthropometric parameters, took standard biochemistry measurements, and screened for complications of diabetes according to the current guideline^16^ (urinary albumin-to-creatinine ratio and serum creatinine concentrations for diabetic kidney disease, retinal photography for diabetic retinopathy, and physical examination and 10 g monofilament examination for diabetic neuropathy). After a two to three day run-in period, participants were admitted to the hospital and received SIIT, as previously described.^13^ Briefly, SIIT was administered using continuous subcutaneous insulin infusion (MiniMed Paradigm 722 insulin pump) to deliver insulin aspart or insulin lispro with an initial total daily insulin dose of 0.5 IU/kg, split 50:50 for basal insulin and pre-meal boluses. Insulin doses were titrated according to capillary blood glucose concentrations, targeting fasting/pre-meal blood glucose of <6.1 mmol/L and two hour postprandial blood glucose of <8.0 mmol/L. On achievement of these targets, insulin infusion was sustained for two weeks. Throughout the hospital stay, participants were supplied with daily diets that followed the current nutritional guidelines and were encouraged to walk or jog for 30-60 minutes after each meal. A nutritionist designed the diet, ensuring that carbohydrates, proteins, and fat accounted for 50-60%, 10-15%, and 20-30%, respectively, of total energy intake. Patients consumed three meals a day, with breakfast, lunch, and dinner accounting for 20%, 40%, and 40% of the total caloric intake, respectively. From the beginning of the hospital stay, participants were provided with guidance on exercise, including recommended speed and duration, and written management tips on a card. Participants walked or jogged in the ward hallway. Healthcare staff monitored and supervised the exercise during daily rounds by discussing participants’ activities, providing further guidance, and recording exercise adherence.

After discontinuation of insulin, baseline measurements were repeated the next morning (at least 15 hours after insulin was stopped). Afterwards, participants received one of the following subsequent therapies according to their assignment: participants randomised to the linagliptin plus metformin group received both linagliptin (5 mg/day; Trajenta, Boehringer-Ingelheim, Germany) and metformin (1000 mg/day; Glucophage, Merck Serono, Switzerland) for 48 weeks; participants in the linagliptin group were treated with linagliptin (5 mg/day) for 48 weeks; participants in the metformin group were treated with metformin (1000 mg/day) for 48 weeks; and participants assigned to the control group received no anti-diabetes medicine after SIIT.

Participants were encouraged to adhere to lifestyle changes and met with study staff every 12 weeks for repeat measurement of the parameters assessed at baseline. During each follow-up visit, we assessed adherence to drug treatment by calculating the number of returned tablets; researchers provided lifestyle change consultations consistent with routine diabetes management practices.^16^ The consultations included a review of the participants’ dietary habits, exercise duration and intensity, weight changes, and glycaemic control, as well as guidance to improve their nutritional habits and maintain at least 150 minutes of moderate intensity physical activity per week. Oral anti-diabetes drugs were held for at least 48 hours before visits at which blood sample were taken. In addition, participants were advised to measure fasting capillary blood glucose at least twice a week and document these values in their patient diaries. Participants were asked to contact study staff if fasting capillary blood glucose exceeded 9.0 mmol/L. If HbA_1c_ exceeded 8.0% after week 12 or fasting blood glucose was above 9.0 mmol/L continuously for two weeks as confirmed by venous blood glucose, the participant was returned to standard clinical care according to the Chinese diabetes guideline.^16^

### Biochemical analyses

Plasma samples were centrally processed at the Kingmed Center for Clinical Laboratory (Guangzhou). HbA_1c_ was measured by high performance liquid chromatography (Bio-Rad, Hercules, CA, USA). Insulin concentrations were determined using chemiluminescent immunoassay (Roche Diagnostics GmbH, Germany). Lipid profiles, transaminase, and serum creatinine were measured at designated visits. A mixed meal tolerance test was accomplished by participants consuming 100 g of commercial instant noodles, with blood samples collected at fasting and 30, 60, and 120 minutes after ingestion of the mixed meal.

We measured insulin sensitivity by using the homoeostasis model assessment (HOMA-IR) and the Matsuda index. We assessed β cell function with the homoeostasis model assessment (HOMA-β) and the insulin secretion-sensitivity index-2 (ISSI-2), which we calculated as the product of the insulin-to-glucose area under the curve ratio (AUC_ins_/AUC_gluc_) and the Matsuda index.^17^ ^18^

### Outcomes

The study’s primary outcome was optimal glycaemic control, predefined as HbA_1c_ <7.0% at 48 weeks after SIIT. Secondary outcomes included the percentage of participants achieving HbA_1c_ <6.5%, changes in HbA_1c_, fasting and two hour postprandial plasma glucose in the mixed meal tolerance test, β cell function indices, and insulin sensitivity indices from baseline. We used capillary blood glucose recorded by patients during follow-up only for monitoring hyperglycaemia relapse and did not include it in the outcome analysis. Frequencies of adverse events were recorded. We classified episodes of hypoglycaemia as level 1 (blood glucose <3.9 mmol/L but ≥3.0 mmol/L), level 2 (blood glucose <3.0 mmol/L), and level 3 (altered mental and/or physical status requiring assistance for correction of hypoglycaemia).

### Statistical analysis

Sample size estimation suggested that 89 participants per group could provide 80% power to detect a 20% higher probability of achieving HbA_1c_ <7.0% in the linagliptin plus metformin group versus the control group at week 48. This calculation assumed that 75% of participants in the intervention group and 55% in the control group would achieve this endpoint with a type I error of 2.5% in the one sided test. Anticipating a 15% dropout rate, we needed 103 per arm (412 in total).

We did analyses by following the intention-to-treat principle. We did multiple imputations within each group for the missing endpoints (HbA_1c_ <7.0% and <6.5% at week 48) by using a logistic model with group, gender, age, baseline body mass index, and HbA_1c_ levels as predictors. We imputed five complete datasets and combined the estimates from each imputed dataset into one overall estimate in SPSS. We did per protocol analyses, as well as sensitivity analyses treating missing 48 week HbA_1c_ data as not achieving the primary endpoint, to assess the robustness of the results.

We expressed continuous variables as mean (standard deviation) for normally distributed data and compared them via one way analysis of variance or as median (interquartile range) for skewed data and compared them via Kruskal-Wallis H tests. For comparisons of trial outcomes among treatment groups, we applied generalised linear models for continuous variables and used the χ^2^ test or Fisher’s exact test for categorical variables. We evaluated longitudinal changes over time in outcome variables by using a generalised estimating equation model, with goodness of fit optimised using the quasi-likelihood under the independence model criterion. We compared Kaplan-Meier curves with log-rank tests and used a logistic regression model to calculate the risk ratio in the primary endpoint between the treatment groups. We considered P values of <0.05 to be statistically significant. To reduce the risk of type I errors in multiple comparisons between the treatment and control groups, we set the significance level at P<0.0167 in pairwise comparison. We used SPSS version 19.0 and GraphPad Prism version 9.0 for statistical procedures.

### Patient and public involvement

Patients or members of the public were not directly involved in the design, conduct, reporting, or analysis of the trial. This trial was started before patient and public involvement gained widespread adoption in research practice, and the necessary funding to facilitate such involvement was not allocated. Nonetheless, the study protocol and treatment strategies underwent extensive consultation with clinical endocrinologists and were rigorously reviewed by the ethics board. Furthermore, clinical endocrinologists will play a key role in communicating the results to patients, the public, and healthcare professionals to encourage the translation of research into practice. Once our paper is published, we plan to publish a press release. We further plan to present the study outcomes at annual meetings of endocrinology societies in China and internationally, hoping the strategy will be adopted by more centres in China and worldwide, which will require further input from the public, including patients.

## Results

### Baseline characteristics and SIIT

**Fig 1:**
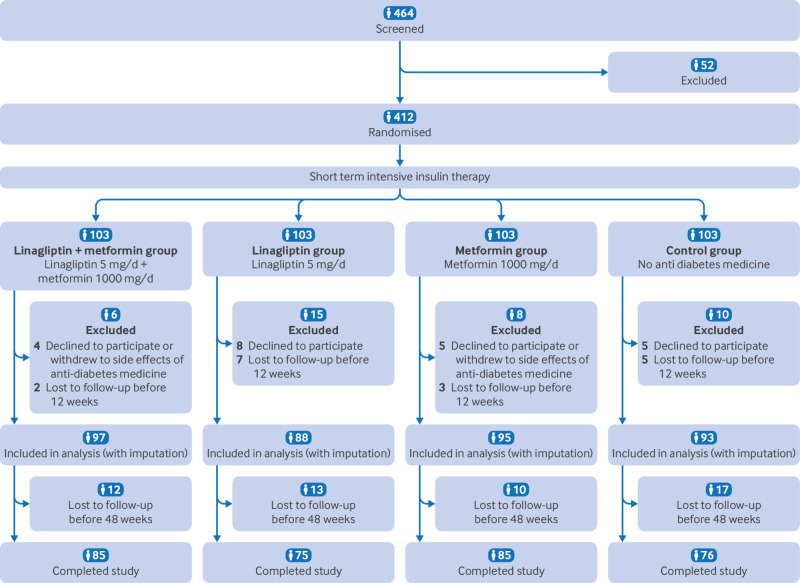
Trial profile

A total of 464 patients were screened between December 2017 and December 2020, of which 412 were eligible and were subsequently randomised and treated with SIIT. Thirty nine participants who did not return for any follow-up after SIIT were excluded from further efficacy analysis. Among the remaining 373 patients (full analysis set), 321 completed the 48 week follow-up (per protocol set), with 12 from the linagliptin plus metformin group, 13 from the linagliptin group, 10 from the metformin group, and 17 from the control group dropping out during follow-up (fig 1).

The baseline characteristics were well balanced across the four groups (intention-to-treat population, n=412; table 1). The participants had an average age of 46.8 (standard deviation (SD) 11.2) years, a mean body mass index of 25.8 (2.9), and an estimated duration of diabetes of 1.0 (interquartile range 0.5-5.0) months. Among the randomised participants, 75% (311/412) had a disease duration of less than six months and 25% (101/412) had a duration of six months or more (up to three years), including 16% (64/412) with a duration of more than one year. All participants had significantly elevated HbA_1c_ (11.0% (SD 1.9%)), fasting plasma glucose (11.4 (2.9) mmol/L), and two hour postprandial glucose (20.5 (4.0) mmol/L) concentrations, with 76% (312/412) of the participants showing symptoms of hyperglycaemia (that is, polyuria, polydipsia, or unexpected weight loss). The initial insulin dose was 37.6 (SD 25.4) IU/day (0.50 (0.35) IU/kg/day). During SIIT, near-normoglycaemia was achieved in 3.8 (SD 2.3) days, with a mean maximum daily insulin dose of 53.4 (18.7) IU/day (0.74 (0.22) IU/kg/day). Afterwards, the insulin dose gradually decreased to 32.0 (SD 15.0) IU/day (0.45 (0.21) IU/kg/day) at the end of SIIT, with no significant differences among the treatment groups. As anticipated, in the overall cohort, fasting glucose decreased from 11.4 (SD 2.9) mmol/L to 5.9 (1.1) mmol/L, two hour postprandial glucose decreased from 20.5 (4.0) mmol/L to 13.8 (3.0) mmol/L, and HbA_1c_ decreased from 11.0% (1.9%) to 9.2% (1.3%) after SIIT (all P<0.001). We observed improvements in lipid profiles and recovery in both β cell function and insulin sensitivity, with no significant differences among the groups.

**Table 1:** Baseline characteristics and effects of short term intensive insulin therapy treatment in four intervention groups. Values are means (standard deviation) unless stated otherwise

Characteristic	Linagliptin plus metformin (n=103)		Linagliptin (n=103)		Metformin (n=103)	Control (n=103)	P value
Age, years	47.4 (11.2)		48.5 (10.7)		45.2 (11.3)	46.2 (11.2)	0.17
Gender:							
Male	75		74		71	83	0.27
Female	28		29		32	20	
Median (IQR) estimated disease duration, months	1.0 (0.6-12.0)		1.0 (0.5-3.0)		1.0 (0.5-5.5)	1.0 (0.5-4.2)	0.68
Systolic blood pressure, mm Hg	130.9 (15.6)		128.8 (16.9)		129.1 (15.1)	130.0 (17.6)	0.82
Diastolic blood pressure, mm Hg	83.3 (11.2)		82.8 (10.6)		82.8 (10.6)	82.7 (11.7)	0.98
Body mass index:							
Before SIIT	25.7 (2.8)		25.7 (3.0)		25.7 (3.0)	26.0 (2.8)	0.88
After SIIT	25.2 (2.8)		25.2 (3.0)		25.4 (2.9)	25.5 (2.8)	0.78
Waist circumference, cm:							
Before SIIT	91.7 (8.0)		92.0 (8.0)		91.1 (9.7)	92.3 (8.2)	0.82
After SIIT	90.7 (7.6)		90.6 (8.0)		90.6 (9.2)	91.4 (8.3)	0.88
Waist-to-hip ratio:							
Before SIIT	0.9 (0.1)		0.9 (0.1)		0.9 (0.1)	0.9 (0.1)	0.74
After SIIT	0.9 (0.1)		0.9 (0.1)		0.9 (0.1)	0.9 (0.1)	0.98
Serum creatinine, μmol/L	65.6 (15.0)		65.2 (14.5)		62.7 (14.3)	67.1 (15.1)	0.19
Alanine transaminase, U/L	31.6 (24.7)		33.9 (28.9)		33.8 (27.3)	33.0 (24.7)	0.92
Cholesterol, mmol/L:							
Before SIIT	5.7 (1.9)		5.3 (1.0)		5.3 (1.3)	5.3 (1.1)	0.11
After SIIT	4.5 (1.0)		4.6 (1.1)		4.6 (0.9)	4.4 (1.0)	0.56
Triglycerides, mmol/L:							
Before SIIT	2.3 (1.7)		2.3 (1.3)		2.5 (2.0)	2.2 (1.5)	0.67
After SIIT	1.3 (0.4)		1.4 (0.6)		1.3 (0.5)	1.3 (0.6)	0.25
High density lipoprotein cholesterol, mmol/L:							
Before SIIT	1.0 (0.4)		1.0 (0.2)		1.1 (0.6)	1.1 (0.5)	0.51
After SIIT	1.1 (0.5)		1.1 (0.3)		1.0 (0.2)	1.1 (0.3)	0.91
Low density lipoprotein cholesterol, mmol/L:							
Before SIIT	3.5 (0.9)		3.4 (0.9)		3.3 (0.9)	3.4 (1.0)	0.36
After SIIT	2.8 (0.8)		2.9 (0.8)		2.9 (0.7)	2.8 (0.9)	0.76
HbA_1c_, %:							
Before SIIT	11.0 (1.7)		10.8 (1.6)		10.9 (1.7)	11.1 (2.2)	0.59
After SIIT	9.2 (1.3)		9.1 (1.2)		9.2 (1.3)	9.3 (1.4)	0.91
Fasting plasma glucose, mmol/L:							
Before SIIT	11.5 (3.0)		11.1 (3.0)		11.7 (2.9)	11.2 (2.8)	0.53
After SIIT	6.0 (1.2)		5.9 (1.1)		5.8 (1.1)	5.8 (1.0)	0.79
2 hour plasma glucose, mmol/L:							
Before SIIT	21.3 (3.9)		20.2 (4.2)		20.1 (3.9)	20.3 (3.9)	0.12
After SIIT	14.2 (2.9)		13.4 (3.3)		13.9 (2.7)	13.9 (3.0)	0.34
Matsuda index (IQR):							
Before SIIT	4.6 (3.4-7.4)		5.0 (3.3-6.9)		4.5 (3.3-6.4)	4.8 (3.3,6.8)	0.76
After SIIT	6.7 (4.9-9.0)		6.2 (4.3-10.4)		6.7 (4.6-10.6)	7.3 (5.3-9.9)	0.48
ISSI-2 (IQR):							
Before SIIT	53.6 (37.4-97.6)		65.4(44.6-103.9)		58.1 (43.3-91.6)	62.8 (44.4-101.6)	0.57
After SIIT	264.9 (197.3-375.6)		258.9 (186.8-370.8)		265.4 (199.1-362.4)	264.5 (199.8-371.6)	0.96
HOMA-IR (IQR):							
Before SIIT	3.2 (2.2-4.3)		3.1 (2.0-4.3)		3.3 (2.3-4.5)	2.9 (2.0-4.8)	0.96
After SIIT	1.3 (0.8-2.0)		1.5 (0.8-2.3)		1.3 (0.9-2.0)	1.2 (0.8-2.0)	0.67
HOMA-β (IQR):							
Before SIIT	15.5 (8.9-26.2)		17.2 (10.9-34.2)		16.3 (10.8-28.4)	14.8 (10.1-31.5)	0.68
After SIIT	43.2 (27.6-71.4)		44.6 (31.0-80.8)		50.0 (31.7-78.3)	42.8 (31.5-68.1)	0.48
No (%) diabetic retinopathy	5 (5)		7 (7)		6 (6)	8 (8)	0.84
No (%) microalbuminuria	5 (5)		3 (3)		6 (6)	8 (8)	0.48

Comparisons of parameters after SIIT have been adjusted for baseline values.

HbA_1c_=glycated haemoglobin A_1c_; HOMA-β=homoeostasis model assessment of β cell function; HOMA-IR=homoeostasis model assessment of insulin sensitivity; IQR=interquartile range; ISSI-2=Insulin Secretion-Sensitivity Index-2; SIIT=short term intensive insulin therapy.

### HbA_1c_ control

**Fig 2:**
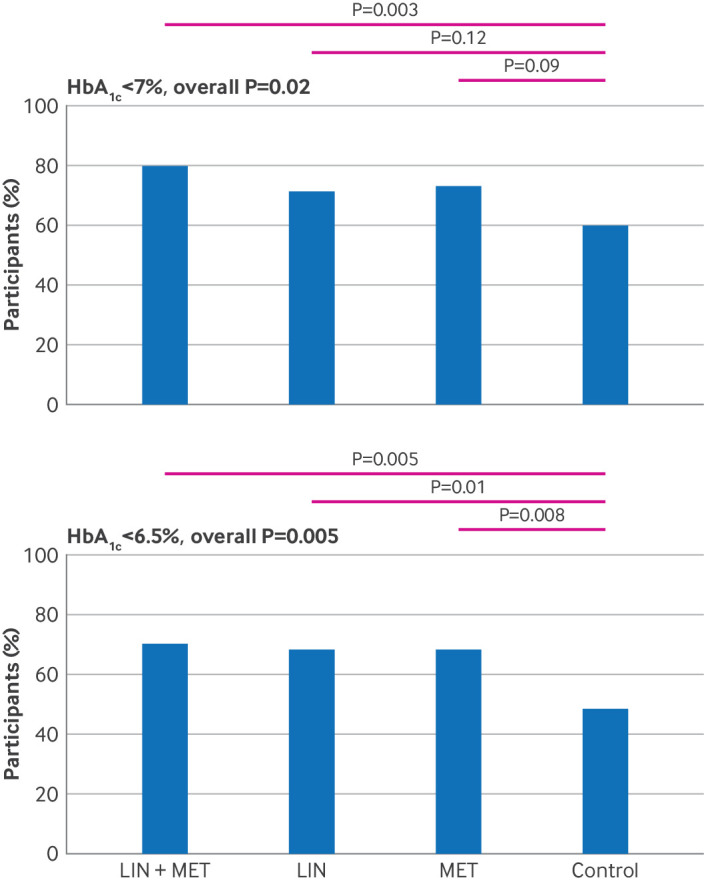
Glycated haemoglobin A_1c_ (HbA_1c_) control during follow-up. Proportions of participants achieving HbA_1c_ <7.0% or HbA_1c_ <6.5% at week 48. LIN=linagliptin group; LIN+MET=linagliptin plus metformin group; MET=metformin group

The interaction between linagliptin and metformin treatment was not significant (P=0.63). We therefore assessed the treatment effects of different subsequent regimens independently. In the full analysis set, we generated five datasets by using multiple imputation and pooled them for HbA_1c_ endpoint analyses (supplementary tables S3 and S4). Overall, 71% (266/373) of participants achieved the primary endpoint of HbA_1c_ <7.0% at week 48. Participants with a disease duration of less than six months tended to have a higher likelihood of achieving this HbA_1c_ target (supplementary table S9). The proportions of participants achieving HbA_1c_ <7.0% at week 48 were 80% (78/97) in the linagliptin plus metformin group, 72% (63/88) in the linagliptin group, 73% (69/95) in the metformin group, and 60% (56/93) in the control group (χ^2^ test: overall P=0.02). After adjustment for multiple comparisons, the difference between the linagliptin plus metformin group and the control group reached statistical significance (P=0.003), whereas the linagliptin group versus the control group and the metformin group versus the control group did not (P=0.12 and P=0.09, respectively) (fig 2). Additionally, 70% (68/97), 68% (60/88), and 68% (65/95) of participants in the linagliptin plus metformin, linagliptin, and metformin groups achieved HbA_1c_ <6.5%, compared with 48% (45/93) in the control group (χ^2^ test: P=0.005 overall; P=0.005 for linagliptin plus metformin versus control; P=0.01 for linagliptin versus control; P=0.008 for metformin versus control; all were significant after adjustment for multiple comparisons) (fig 2). Logistic analysis, with trial centres set as a random effect, indicated that compared with the control group, the linagliptin plus metformin group (odds ratio 2.78, 95% confidence interval (CI) 1.37 to 5.65; P=0.005) was more likely to achieve the HbA_1c_ <7.0% endpoint after adjustment for age, gender, body mass index, and baseline HbA_1c_, whereas the relative effects in the linagliptin group (1.70, 0.84 to 3.42; P=0.17) and the metformin group (1.77, 0.91 to 3.42; P=0.07) were not significant. We found similar results in the sensitivity analysis and the per protocol analysis (supplementary figure S1). In the per protocol analysis, the proportions of participants achieving HbA_1c_ <7% at 48 weeks were 86% (73/85) in the linagliptin plus metformin group, 79% (59/75) in the linagliptin group, 76% (65/85) in the metformin group, and 63% (48/76) in the control group (χ^2^ test: overall P=0.008). The difference was significant between the linagliptin plus metformin group and the control group (P<0.001) but not significant for the linagliptin group or metformin group versus the control group after adjustment for multiple comparisons (P=0.05 and P=0.08, respectively).

**Fig 3:**
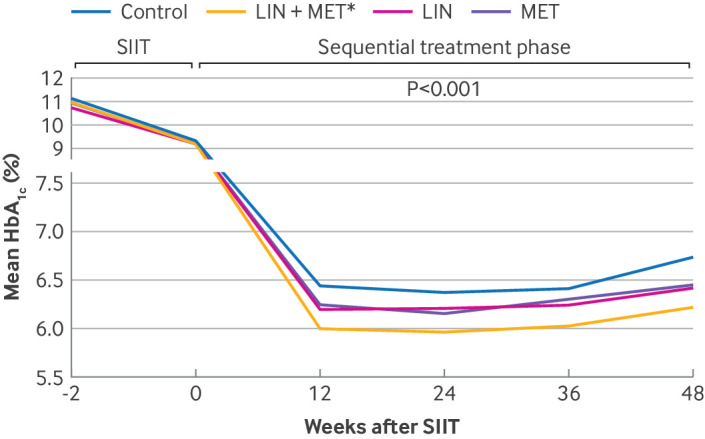
Glycated haemoglobin A_1c_ (HbA_1c_) control during follow-up. Change in HbA_1c_ over time. LIN=linagliptin group; LIN+MET=linagliptin plus metformin group; MET=metformin group; SIIT=short term intensive insulin therapy. *P<0.0167 compared with control group in log-rank tests

**Fig 4:**
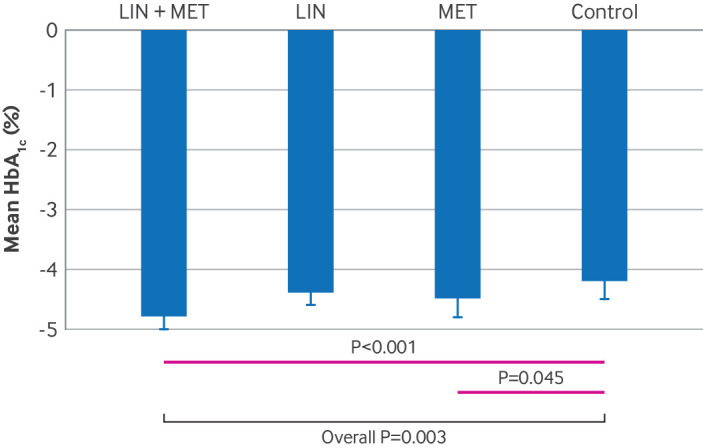
Glycated haemoglobin A_1c_ (HbA_1c_) control during follow-up. Change in HbA_1c_ at week 48 from baseline. LIN=linagliptin group; LIN+MET=linagliptin plus metformin group; MET=metformin group

As shown in figure 3, the HbA_1c_ level was significantly lower in the linagliptin plus metformin group across the follow-up period. At week 48, the HbA_1c_ levels were 6.2% (95% CI 6.0% to 6.4%) in the linagliptin plus metformin group, 6.5% (6.3% to 6.7%) in the linagliptin group, 6.4% (6.2% to 6.6%) in the metformin group, and 6.7% (6.5% to 6.9%) in the control group, after adjustment for age, gender, body mass index, and baseline HbA_1c_ (P=0.004 overall; P<0.001 for linagliptin plus metformin versus control; P=0.02 for linagliptin plus metformin versus linagliptin; P=0.05 for metformin versus control group) (table 2). The mean change in HbA_1c_ from baseline was −4.8% (95% CI −5.0% to −4.6%) in the linagliptin plus metformin group, −4.4% (−4.6% to −4.2%) in the linagliptin group, −4.5% (−4.8% to −4.3%) in the metformin group, and −4.2% (−4.5% to −4.0%) in the control group (overall P=0.003) (fig 4). We noted significant differences between the linagliptin plus metformin group and the control group (P<0.001) and between the metformin group and the control group (adjusted P=0.045) (table 2).

**Table 2:** Clinical outcomes at 48 week follow-up. Values are least squares mean (95% confidence interval) unless stated otherwise

Outcome	Linagliptin plus metformin (n=97)	Linagliptin (n=88)	Metformin (n=95)	Control (n=93)	P value
HbA_1c_, %:					
At week 48	6.2 (6.0 to 6.4)*	6.5 (6.3 to 6.7)*	6.4 (6.2 to 6.6)*	6.7 (6.5 to 6.9)	0.004
Change from baseline	−4.8 (−5.0 to −4.6)*	−4.4 (−4.6 to −4.2)	−4.5 (−4.8 to −4.3)*	−4.2 (−4.5 to −4.0)	0.003
Fasting plasma glucose, mmol/L:					
At week 48	6.0 (5.6 to 6.3)*	6.8 (6.4 to 7.2)	6.5 (6.1 to 6.9)	6.6 (6.2 to 6.9)	0.01
Change from baseline	−5.3 (−4.9 to −5.7)*	−4.5 (−4.1 to −4.8)	−4.8 (−4.4 to −5.1)	−4.7 (−4.3 to −5.1)	0.008
2 hour plasma glucose, mmol/L:					
At week 48	10.5 (9.7 to 11.2)	10.6 (9.8 to 11.3)	11.6 (10.9 to 12.4)	11.3 (10.5 to 12.2)	0.09
Change from baseline	−10.7 (−11.9 to −9.6)*	−9.5 (−10.7 to −8.4)	−8.5 (−9.7 to −7.4)	−8.3 (−9.6 to −7.0)	0.14
Matsuda index:					
At week 48	5.9 (5.1 to 6.5)	4.8 (4.1 to 5.5)	4.6 (4.0 to 5.4)	5.5 (4.7 to 6.3)	0.06
Change from baseline	0.5 (−0.25 to 1.3)	−0.3 (−1.1 to 0.5)	−0.5 (−1.3 to 0.3)	0.3 (−0.6 to 0.3)	0.21
ISSI-2:					
At week 48	345.7 (310.4 to 379.0)*	287.8 (252.5 to 323.0)	280.1 (241.7 to 318.4)	265.4 (231.4 to 299.5)	0.005
Change from baseline	273.1 (237.6 to 308.6)*	207.2 (170.6 to 243.7)	190.9 (155.6 to 226.2)	193.1 (153.4 to 232.9)	0.002
HOMA-IR:					
At week 48	2.7 (2.2 to 3.3)	4.0 (3.5 to 4.5)	3.3 (2.8 to 3.8)	3.2 (2.7 to 3.8)	0.006
Change from baseline	−0.6 (−1.3 to 0.0)	0.3 (−0.3 to 1.0)	−0.5 (−1.1 to 0.1)	−0.4 (−1.1 to 0.2)	0.11
HOMA-β:					
At week 48	103.1 (85.6 to 120.7)	101.9 (83.9 to 120.0)	95.5 (78.1 to 112.9)	79.4 (59.8 to 99.1)	0.24
Change from baseline	81.0 (64.0 to 98.0)	77.9 (60.4 to 95.4)	72.5 (55.6 to 89.4)	52.3 (33.2 to 71.3)	0.10

Comparisons were adjusted for age, gender, body mass index, and baseline HbA_1c_.

HbA_1c_=glycated haemoglobin A_1c_; ISSI-2=Insulin Secretion-Sensitivity Index-2; HOMA-β=homoeostasis model assessment of β cell function; HOMA-IR=homoeostasis model assessment of insulin resistance.

* Adjusted P<0.05 compared with control group.

**Fig 5:**
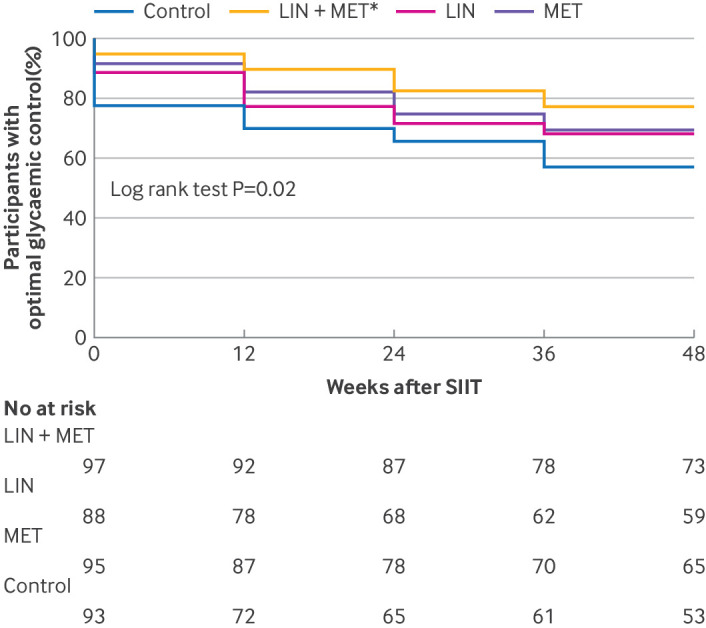
Glycated haemoglobin A_1c_ (HbA_1c_) control during follow-up. Summarisation of participants in optimal glycaemic control using Kaplan-Meier curves. LIN=linagliptin group; LIN+MET=linagliptin plus metformin group; MET=metformin group; SIIT=short term intensive insulin therapy. *P<0.0167 compared with control group in log-rank tests

We did a survival analysis to explore the time to loss of optimal glycaemic control, defined as HbA_1c_ ≥7.0% since week 12 or fasting plasma glucose ≥7.0 mmol/L between the cessation of SIIT and week 12. The linagliptin plus metformin group had a significantly higher probability of maintaining optimal glycaemic control than the control group (log-rank test: P=0.001). After adjustment for multiple comparisons, the comparison between the linagliptin plus metformin group and the control group did not reach statistical significance (log-rank test: P=0.09 and P=0.04, respectively) (fig 5).

### Other outcomes

**Fig 6:**
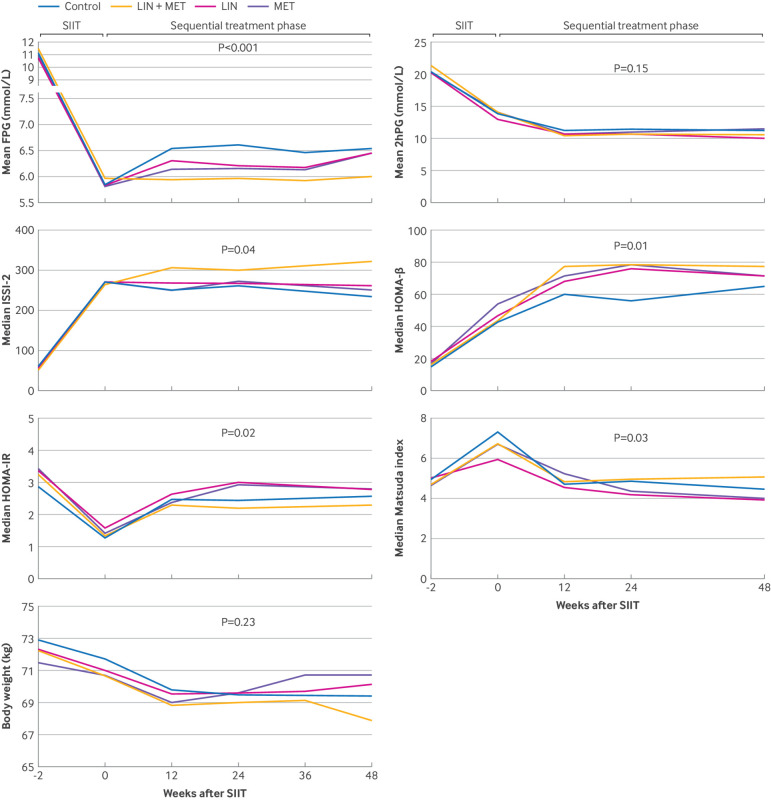
Changes of clinical parameters over time in different treatment groups. Variables are compared with generalised estimating equation models adjusted for age, gender, baseline body mass index, and baseline HbA_1c_. P<0.0167 compared with control group for LIN+MET in mean FPG and ISSI-2 and for all treatment groups in HOMA-β. 2HPG=two hour postprandial glucose; FPG=fasting plasma glucose; HOMA-β=homoeostasis model assessment of β cell function; HOMA-IR=homoeostasis model assessment of insulin resistance; ISSI-2=insulin secretion-sensitivity index-2; LIN=linagliptin group; MET=metformin group; LIN+MET=linagliptin plus metformin group; SIIT=short term intensive insulin therapy

The changes in other outcomes are summarised in figure 6 and table 2. As shown in fig 6 (top left), the subsequent treatment groups maintained stable fasting plasma glucose concentrations throughout the follow-up period, particularly in the linagliptin plus metformin group. At week 48, the linagliptin plus metformin group had the lowest fasting plasma glucose concentrations and the greatest reduction in fasting plasma glucose from baseline. The mean two hour postprandial glucose concentrations did not differ among treatment groups (fig 6, top right). The linagliptin plus metformin group had better preservation of ISSI-2 than did other groups throughout the follow-up period (generalised estimating equation model analyses: P=0.04 overall; P=0.001 for the linagliptin plus metformin group versus the control group) (fig 6, second graph on left). At week 48, mean ISSI-2 values were 345.7 (95% CI 310.4 to 379.0), 287.8 (252.5 to 323.0), 280.1 (241.7 to 318.4), and 265.4 (231.4 to 299.5) in the linagliptin plus metformin, linagliptin, metformin, and control groups, respectively (P=0.009 for linagliptin plus metformin versus control) (table 2). Similarly, the three subsequent treatment groups showed significantly higher HOMA-β values than did the control group throughout the follow-up until the end of the study (fig 6, middle right). Insulin sensitivity variables—HOMA-IR and Matsuda index—showed similar patterns of change over time (fig 6, third left and bottom right). In generalised estimating equation model analyses, the between group differences in HOMA-IR and Matsuda index were both significant (P=0.02 and 0.03, respectively). However, after adjustment for multiple comparisons, none of the comparisons between the three subsequent treatment groups and the control group was significant (all P>0.0167). We observed a mild reduction in body weight in all treatment groups (−2.0 (interquartile range −4.7-0.5 kg); −3.5% (SD 0.7%) of baseline body weight), with no significant inter-group difference (P=0.23) (fig 6, bottom left). The medication adherence rates for the linagliptin plus metformin, linagliptin, and metformin groups were 87.7% (SD 5.4%), 92.2% (4.4%), and 91.0% (3.9%), respectively.

### Safety

During the SIIT phase, an average of 2.0 (SD 2.6) episodes of level 1 hypoglycaemia and 0.15 (0.46) episodes of level 2 hypoglycaemia occurred in each patient, neither with significant differences between groups. No episodes of severe hypoglycaemia occurred during either SIIT or subsequent therapies. A total of 41 participants needed additional glucose lowering interventions during the follow-up period owing to relapse of hyperglycaemia reaching the previously described thresholds (fasting plasma glucose >9.0 mmol/L or HbA_1c_ >8.0%; seven in the linagliptin plus metformin group, 10 in the linagliptin group, nine in the metformin group, and 15 in the control group; supplementary table S8). At the time of additional intervention, the mean HbA_1c_ was 8.0% (SD 0.6%) and the mean fasting plasma glucose was 9.3 (1.1) mmol/L. Of these hyperglycaemic relapse events, 73% (30/41) occurred within 24 weeks after SIIT treatment, and 22% (9/41) of the patients with a hyperglycaemic relapse received injectable therapy (insulin or glucagon-like peptide 1 receptor agonists). Adverse events during the subsequent therapy period are summarised in supplementary table S5. Overall, the subsequent oral regimens were well tolerated. The most common adverse events were gastrointestinal side effects, more prevalent in the metformin group (16/103; 16%) and the linagliptin plus metformin group (18/103; 17%) than in the linagliptin group (7/103; 7%) and the control group (4/103; 4%). Withdrawal due to gastrointestinal side effects occurred in three participants from the linagliptin plus metformin group and five from the metformin group. No participant died.

## Discussion

In this proof-of-principle study, the implementation of the intense simplified strategy significantly improved glycaemic outcomes. SIIT rapidly eliminated glucotoxicity (mean fasting glucose decreased from 11.4 mmol/L to 5.9 mmol/L) and improved β cell function, and these glycaemic benefits were well maintained with simple oral medication regimens. Over 48 weeks, 80.4%, 71.6%, and 72.6% of patients in the linagliptin plus metformin, linagliptin, and metformin groups, respectively, achieved HbA_1c_ <7.0%. These rates were even higher in the per protocol analysis (85.9%, 78.7%, and 76.5%, respectively; supplementary figure S1). These favourable outcomes were accompanied by more stable β cell recovery. The findings suggest that this intense simplified strategy merits further consideration in managing early diabetes in which significant hyperglycaemia is noted.

### Potential rationale

As previously stated, SIIT exerts its effect uniquely by rapidly clearing glucotoxicity and lipotoxicity. Previous studies have shown that rigorous glycaemic control during SIIT, characterised by lower mean blood glucose and higher time in range within predefined glycaemic targets, is crucial for favourable outcomes.^19^ ^20^ This is supported by basic experimental data, as stringent glycaemic control restored the response pattern to glucose in human β cells and induced β cell redifferentiation.^21^ ^22^ However, maintaining these benefits over time is challenging. Hyperglycaemic relapse may occur as a result of ageing, poor adherence to diabetes management, and insufficient intervention for underlying mechanisms of type 2 diabetes mellitus, such as impaired incretin action.^23^ ^24^ Notably, even mild hyperglycaemia can result in β cell impairment,^25^ ^26^ potentially exacerbating a vicious cycle. Therefore, sequentially applying therapies targeting multiple mechanisms of type 2 diabetes after SIIT may be essential for maintaining durable glycaemic control and preventing functional β cell loss.

### Comparison with other studies

This study of the intense simplified strategy substantially differs from several previous studies investigating the effect of early initiation of combination therapy on reducing treatment failure in newly diagnosed type 2 diabetes. In the VERIFY study, vildagliptin plus metformin reduced the risk of initial treatment failure (hazard ratio 0.51). However, the VERIFY study enrolled only patients with mild hyperglycaemia (HbA_1c_ 6.5-7.5%).^27^ In a randomised controlled trial, linagliptin plus high dose metformin (up to 2000 mg/day) reduced HbA_1c_ by 2.8% (SD 0.1%) and achieved HbA_1c_ <7.0% in 60.3% of participants with a baseline HbA_1c_ of 10%, but in a shorter observation period (24 weeks).^28^ Another analysis combining data from two randomised controlled trials found that linagliptin plus metformin (1000 mg/day) reduced HbA_1c_ by 1.67% in patients with early type 2 diabetes and moderate hyperglycaemia (mean baseline HbA_1c_ 8.7%), with only 51.4% achieving HbA_1c_ <7.0% and 40.1% achieving HbA_1c_ <6.5%.^29^ Compared with these studies, our study achieved more prominent (mean reduction of HbA_1c_ ranging from 4.2% to 4.8% in the subsequent treatment groups) and durable (48 weeks) optimal glycaemic control in patients with more marked hyperglycaemia (mean HbA_1c_ 11.0%). In the EDICT study in patients with new onset of type 2 diabetes and significant hyperglycaemia (average HbA_1c_ ~11.0%), initial triple therapy, comprising metformin, pioglitazone, and exenatide, achieved HbA_1c_ <6.5% in 78% of participants over three years.^30^ However, long term triple therapy, particularly involving injectable agents, may encounter challenges of adherence, tolerance, and economic pressure.^31^ The first step of intensive treatment lays the foundation for subsequent management by rescuing damaged β cells from persistent metabolic distress (disease modifying property), which allows for achievement of sustained glycaemic control with a convenient medication regimen (linagliptin once daily, moderate dose metformin (1000 mg/day), or a combination). Use of simplified regimens contributed to good drug adherence and tolerability, with withdrawals due to side effects occurring in less than 5% of participants. Moreover, the benefits of the intense simplified strategy were not dependent on weight loss, with participants losing only around 2 kg of body weight over the treatment period. Taken together, the intense simplified strategy provides an effective, safe, and affordable approach for patients with newly diagnosed type 2 diabetes and significant hyperglycaemia, avoiding the disadvantages of complex regimens.^32^ ^33^ This finding is supported by an observational study from Shambo and colleagues, which showed long term glycaemic control and reduced need for anti-diabetes agents after early insulin initiation in type 2 diabetes.^34^

### Limitations of study

This study has some limitations. The intervention was not blinded. Nevertheless, the fact that the primary and secondary endpoints were generated from biochemical measurements by blinded technical staff reduces potential bias. Furthermore, the attrition rate was higher than expected, largely owing to the covid-19 pandemic in China during the later stages of the study, which caused some patients to drop out owing to strict social control measures and travel restrictions. However, on the basis of the number of participants in the full analysis set (97 in the linagliptin plus metformin group and 93 in the control group) and primary endpoint data (80.4% and 60.2%, respectively), the statistical power exceeded 85%. Additionally, sensitivity analyses and per protocol analyses confirmed the robustness of the conclusions. Lastly, although the participants in this study were recruited from 15 centres across China, the findings need to be validated in more diverse national and ethnic populations.

### Conclusions

The intense simplified strategy using subsequent oral therapies after SIIT, especially the linagliptin plus metformin combination, sustainably improved glycaemic control and β cell function in patients with newly diagnosed type 2 diabetes and severe hyperglycaemia. This proof-of-principle study also provides a new area for future exploration. More practical and feasible intensive approaches that can be applied in outpatient settings, such as multiple daily insulin injections or even regimens based on basal insulin, could be explored for the “intense” module, and more convenient drugs such as fixed dose combination preparations or sodium-glucose co-transporter 2 inhibitors with benefits for complications could also be considered in the “simplified” module. Further data are needed to evaluate the durability of treatment effects, the strategy’s impact on reducing long term complications, and its potential economic advantages.

## What is already known on this topic

The optimal management strategy for patients with newly diagnosed type 2 diabetes mellitus and significant hyperglycaemia remains unclear
Short term intensive insulin therapy (SIIT) can induce β cell recovery and remission of diabetes in these patients, but the effects diminish over time
The efficacy of a simplifying strategy after SIIT has not been investigated in randomised controlled trials

## What this study adds

The intense simplified strategy, particularly using linagliptin plus metformin after SIIT, led to excellent glycaemic control in participants with severe hyperglycaemia
This study provides information for establishing a de-escalation therapeutic strategy for patients with newly diagnosed type 2 diabetes and severe hyperglycaemia

## Data Availability

Data from this study can be requested from the corresponding author (liyb@mail.sysu.edu.cn) after its publication. Requests can be made for de-identified participant data, the data dictionary, and other specified datasets. Additionally, the study protocol, statistical analysis plan, and informed consent form can be provided on request. Specific requests for data will require the submission of a proposal with a valuable research question as assessed by the study steering committee and may require a data access agreement to be signed.

## Web extra

Extra material supplied by authors

Web appendix: Supplementary materials

## Notes

Contributors: LL, WK, HL, FL, GF, JK, and JM contributed equally to this work. YL and YM contributed to the conception and design of the study; YL, LL,WK, HL, FL, GF, JK, JM, XZ, BJ, SL, YD, YX, ZL, LG, SQ, YS, LY, WD, ZX, PD, LX, JL, XW, GW, SY, SH, PZ, ZL, XC, ZL, and HX contributed to the execution of the trial. LL, WK, HL, FL, GF, JK, JM, and YH contributed to the collection and analysis of data. YL and LL accessed and verified the underlying data. LL, YL, YH, WK, and HL contributed to drafting and revising the manuscript. All authors critically reviewed drafts of the manuscript and approved the final version. YL, YH, and YM are the guarantors. The corresponding author attests that all listed authors meet authorship criteria and that no others meeting the criteria have been omitted.

Transparency: The lead authors (the manuscript’s guarantors) affirm that the manuscript is an honest, accurate, and transparent account of the study being reported; that no important aspects of the study have been omitted; and that any discrepancies from the study as planned (and, if relevant, registered) have been explained.

Dissemination to participants and related patient and public communities: A lay summary of the results of the study will be shared with participants. Once our paper is published, we plan to publish a press release. We further plan to present the study outcomes at annual meetings of endocrinology societies in China and internationally, hoping the strategy will be adopted by more centres in China and worldwide, which will require further input from the public, including patients.

Provenance and peer review: Not commissioned; externally peer reviewed.
