# Supplementary material for: Intense simplified strategy for newly diagnosed type 2 diabetes in patients with severe hyperglycaemia: multicentre, open label, randomised trial
Source: BMJ. 2024 Oct 15;387:e080122. doi: 10.1136/bmj-2024-080122 (PMC11474422; doi:10.1136/bmj-2024-080122)
Supplement: Supplementary file 1 — Web appendix: Supplementary materials [file liul080122.ww1.pdf]

Supplementary material

1. Statistical analysis of the per-protocol set (PPS)（n=321）

1.1 Baseline and post-SIIT characteristics

The PPS set included 321 participants. The baseline characteristics and treatment effects of SIIT are summarized in Table S1. Baseline variables were well-balanced among the treatment groups. After SIIT, FPG decreased from 11.3±3.0 mmol/L to 5.9±1.1 mmol/L; 2-hour postprandial glucose decreased from 20.4±4.1 mmol/L to 13.7±3.9 mmol/L; and HbA1c decreased from 10.9±1.9% to 9.2±1.4%. β-cell function and insulin sensitivity significantly improved. There were no significant differences in post-treatment parameters between the groups.

	LIN + MET	LIN	MET	Control	P
No.	85	75	85	76	
Age (years)	47.8±10.7	48.9±10.4	44.8±10.9	46.9±10.7	0.09
Gender					
male	61	56	58	63	
Female	24	19	27	13	0.18
Estimated disease duration (months)	1.0 (0.5, 6.0)	1.0 (0.5, 2.0)	1.0 (0.5, 5.5)	1.0 (0.5, 6.0)	0.63
SBP (mmHg)	131.7±16.3	128.8±16.3	130.2±15.2	130.6±18.6	0.76
DBP (mmHg)	84.0±11.3	83.6±10.2	82.7±10.7	82.9±12.0	0.86
BMI (kg/m^2)					
Before SIIT	25.6±2.7	26.0±3.1	25.6±2.7	25.6±2.4	0.68
After SIIT	25.1±2.6	25.5±3.0	25.2±2.6	25.2±2.5	0.19
Waist Circumference (cm)					
Before SIIT	91.6±7.9	93.1±8.0	91.0±9.2	91.5±7.8	0.44
After SIIT	90.4±7.6	91.4±7.7	89.9±8.6	90.5±8.1	0.47
Waist-to-hip ratio					
Before SIIT	0.9±0.1	0.9±0.1	0.9±0.1	0.9±0.1	0.39

After SIIT	0.9±0.1	0.9±0.1	0.9±0.1	0.9±0.1	0.54
Serum creatinine (μmol/L)	65.3±15.3	65.7±14.7	63.4±14.5	68.1±15.9	0.27
Alanine transaminase (U/L)	30.6±23.9	36.4±31.7	36.1±29.0	32.1±26.2	0.45
Cholesterol (mmol/L)					
Before SIIT	5.7±2.1	5.3±1.0	5.5±1.4	5.2±1.0	0.21
After SIIT	4.6±1.1	4.6±1.1	4.6±0.9	4.3±1.0	0.49
Triglyceride (mmol/L)					
Before SIIT	2.2±1.6	2.3±1.3	2.5±2.0	2.3±1.6	0.77
After SIIT	1.3±0.4	1.4±0.6	1.3±0.5	1.3±0.7	0.17
HDL-C (mmol/L)					
Before SIIT	1.0±0.4	1.0±0.2	1.1±0.6	1.1±0.6	0.51
After SIIT	1.1±0.5	1.1±0.4	1.0± 0.2	1.0±0.3	0.32
LDL-C (mmol/L)					
Before SIIT	3.5±0.9	3.4±0.9	3.3±0.9	3.4±1.0	0.61
After SIIT	2.9±0.8	2.9±0.9	2.9±0.6	2.7±0.8	0.28
HbA1c (%)					
Before SIIT	10.9±1.7	10.7±1.7	10.9±1.6	11.1±2.3	0.52
After SIIT	9.2±1.4	9.1±1.3	9.2±1.2	9.3±1.5	0.95
FPG (mmol/L)					
Before SIIT	11.3±2.9	10.9±2.9	11.5±2.7	11.2±3.1	0.56
After SIIT	5.9±1.3	5.8±1.0	5.7±1.0	6.0±1.1	0.70
2hPG (mmol/L)					
Before SIIT	21.2±4.0	19.9±4.2	20.1±4.0	20.2±4.1	0.17
After SIIT	14.3±2.8	12.8±3.1	13.6±2.7	14.0±2.9	0.55
Matsuda index					
Before SIIT	4.7 (3.4, 7.4)	4.8 (3.2, 6.9)	4.5 (3.1, 6.2)	4.6 (3.1, 6.6)	0.74
After SIIT	6.8 (4.9,8.9)	5.9 (4.0, 10.7)	6.4 (4.7, 10.5)	7.1 (5.0, 9.6)	0.90

ISSI-2					
Before SIIT	58.6 (36.7, 101.4)	65.4 (46.2, 106.4)	58.6 (42.6, 94.6)	64.6 (42.6, 110.3)	0.48
After SIIT	264.9(202.7,374.5)	274.9(198.8,383.6)	274.5(208.7,368.1)	257.8(197.1,350.3)	0.92
HOMA-IR					
Before SIIT	3.2(2.1, 4.1)	3.2 (1.9, 4.4)	3.5 (2.3, 4.8)	3.2 (2.0, 5.0)	0.61
After SIIT	1.2(0.8, 1.9)	1.5 (0.8, 2.4)	1.3 (0.8, 2.0)	1.3 (0.9, 2.0)	0.68
HOMA -β					
Before SIIT	17.3 (9.0, 27.2)	18.5 (11.7, 34.7)	16.8 (11.3, 31.4)	17.0 (10.8, 32.6)	0.58
After SIIT	41.3 (27.8, 71.8)	46.2 (34.4, 80.7)	54.3 (32.9, 80.5)	41.6 (31.1, 68.2)	0.57

Table S1. Baseline characteristics and short-term intensive insulin therapy treatment effects in the four intervention groups of PPS. Data were presented as mean ± standard deviation for normally distributed data or as median (interquartile range) for non-normally distributed data. SBP, systolic blood pressure; DBP, diastolic blood pressure; BMI, body mass index; HDL-C, high-density lipoprotein-cholesterol; LDL-C, low-density lipoprotein-cholesterol; HbA1c, glycated haemoglobin A1c; FPG, fasting plasma glucose; 2hPG, 2h plasma glucose; HOMA-IR, homeostasis model assessment of insulin resistance; HOMA-β, homeostasis model assessment of β cell function; ISSI-2, Insulin Secretion-Sensitivity Index-2; SIIT, short-term intensive insulin therapy. The comparisons of parameters after SIIT have been adjusted for baseline values.

1.2 Analysis of the HbA1c endpoints

The proportion of participants achieving the HbA1c target of <7.0% at week 48 was 63.2% (48/76) in the control group, 85.9% (73/85) in group LIN + MET, 78.7% (59/75) in group LIN, and 76.5% (65/85) in group MET (chi-square test over all P=0.008; LIN + MET, LIN, MET vs Control: P<0.001, P=0.05, and P=0.08, respectively). Additionally, 72.9% (62/85), 69.3% (52/75), and 68.2% (58/85) of participants in the LIN + MET, LIN, and MET groups achieved an HbA1c of <6.5%, compared with 46.1% (35/76) in the control group (Overall P=0.001; for LIN + MET vs control, P <0.001; for LIN vs control or MET vs control, P=0.004). Logistic analysis, with trial centres set as a random effect, indicated that compared to the control group, the LIN + MET group (OR 3.57, 95% CI 1.64 to 7.78) and the LIN group (OR 2.18, 95% CI 1.04 to 4.55) were more likely to achieve the HbA1c <7.0% endpoint, while the relative effect in the MET group did not reach significance (OR 1.88, 95% CI 0.92

to 3.84), after adjusting for age, gender, BMI, and baseline HbA1c levels (Figure S1).

Survival analysis showed that the time to lose optimal glycaemic control, defined by HbA1c ≥7.0% since week 12 or FPG ≥7.0mmol between the cessation of SIIT and week 12. The LIN + MET group displayed a significantly higher probability of maintaining optimal glycaemic control when compared with the control group (log-rank test P <0.001).

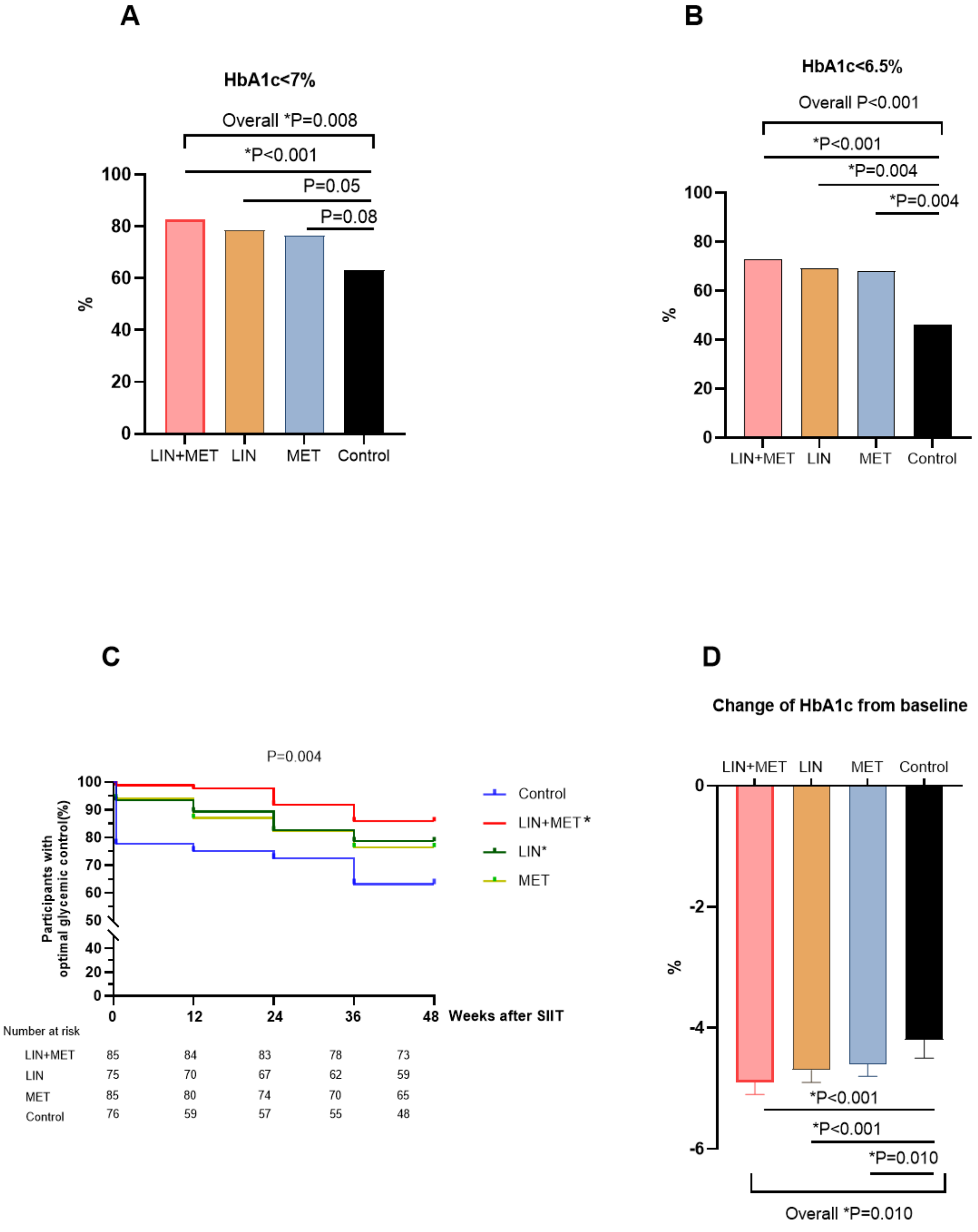

Figure S1. Glycated haemoglobin A1c control during follow-up. The proportions of participants achieving HbA1c <7% or HbA1c <6.5% at week 48 are shown in Figures A and B, respectively; Figure C, changes of proportion of participants with optimal glycaemic control overtime was

summarized with Kaplan-Meier curves,* P <0.0167 compared with the control group in log-rank tests; Figure D, the change of HbA1c from baseline at week 48.

1.3 Other outcomes

Other outcomes, including glycaemic parameters, β cell function indices and insulin sensitivity parameters at week 48 and their change from baseline, were compared in table S2. The results and inter-group differences are consistent with those observed in the FAS analysis.

	LIN+MET	LIN	MET	Control	P-value
No.	85	75	85	76	
HbA1c (%)					
At week 48	6.0 (5.8 to 6.2) *	6.2 (6.0 to 6.4) *	6.3 (6.1 to 6.5) *	6.6 (6.4 to 6.8)	<0.001
Change from baseline	-4.9 (-5.1 to -4.7) *	-4.7 (-4.9 to -4.5) *	-4.6 (-4.8 to -4.4) *	-4.2 (-4.5 to -4.1)	<0.001
FPG (mmol/L)					
At week 48	6.0 (5.8 to 6.2) *	6.2 (6.0 to 6.4)	6.3 (6.1 to 6.5)	6.6 (6.4 to 6.8)	0.04
Change from baseline	-5.4 (-4.7 to -6.0)	-4.5 (-3.9 to -5.2)	-5.1 (-4.4 to -5.7)	-4.3 (-3.6 to -5.1)	0.13
2hPG (mmol/L)					
At week 48	10.4 (9.7 to 11.1)	10.0 (9.3 to 10.7) *	11.5 (10.8 to 12.2)	11.3 (10.6 to 12.1)	0.008
Change from baseline	-10.8 (-11.9 to -9.8) *	-10.3 (-11.4 to -9.2) *	-8.7 (-9.8 to -7.6)	-8.4 (-9.6 to -7.2)	0.006
Matsuda index					
At week 48	5.9 (5.2 to 6.6)	4.9 (4.1 to 5.6)	4.7 (4.0 to 5.4)	5.6 (4.8 to 6.3)	0.06
Change from baseline	0.4 (-0.3 to 1.2)	-0.4 (-1.2 to 0.4)	-0.6 (-1.6 to 0.2)	0.2 (-0.6 to 1.0)	0.21
ISSI-2					
At week 48	346.8 (313.7 to 379.9) *	300.4 (265.9 to 334.9)	268.3 (234.5 to 302.0)	282.1 (CI 245.6 to 318.6)	0.008
Change from baseline	274.6 (240.2 to 308.9) *	219.2 (183.4 to 255.0)	193.0 (158.0 to 228.1)	194.7 (156.8 to 232.5)	0.05

HOMA-IR					
At week 48	2.6 (2.1 to 3.1)	3.8 (3.3 to 4.3)	3.2 (2.7 to 3.7)	3.1 (2.6 to 3.7)	0.01
Change from baseline	-0.7 (-1.3 to 0)	0.3 (-0.4 to 0.9)	-0.5 (-1.1 to 0.1)	-0.4 (-1.1 to -0.2)	0.19
HOMA-β					
At week 48	100.4 (83.3 to 117.5)	102.4 (84.6 to 120.3)	92.9 (75.5 to 110.4)	76.7 (57.8 to 95.5)	0.20
Change from baseline	78.8 (62.2 to 95.4)	78.8 (61.5 to 96.1)	70.5 (53.6 to 87.4)	50.1 (31.9 to 68.4)	0.09

Table S2. Clinical outcomes at 48-week follow-up. HbA1c, glycated hemoglobin A1c; FPG, fasting plasma glucose; 2hPG, 2h plasma glucose; ISSI-2, Insulin Secretion-Sensitivity Index-2; HOMA-IR, homeostasis model assessment of insulin resistance; HOMA-β, homeostasis model assessment of β cell function; *, adjusted P <0.05 compared with the control group. Data are presented as L.S. mean (95% CI). Comparisons were adjusted for age, gender, body mass index and baseline HbA1c.

2. Details of multiple imputation analyses and sensitivity analyses

2.1 Multiple imputation

Using a logistic model, multiple imputations were performed for the missing HbA1c levels to evaluate the endpoints of HbA1c <7.0% and <6.5% at week 48. Missing values were imputed within each group, adjusting for gender, age, baseline BMI, and HbA1c levels. Five datasets were generated for the analysis of HbA1c <7.0% and HbA1c <6.5% at week 48. The results of individual dataset and pool of these results are shown in Table S3 and Table S4. In addition to the data presented in the main text, we performed a logistic regression analysis using week 48 HbA1c <6.5% as the dependent variable. Compared to the control group, all treatment groups showed a higher possiblity of achieving HbA1c <6.5% at week 48 (OR for LIN+MET group: 2.778, 95% CI: 1.431 to 5.125, P=0.005; OR for LIN group: 2.571, 95% CI: 1.363 to 4.850, P=0.004; OR for MET group: 2.480, 95% CI: 1.289 to 4.772, P=0.007).

Datasets	LIN+MET	LIN	MET	Control	P
1	80.4% (78/97)	70.5% (62/88)	74.7% (71/95)	62.4% (58/93)	0.04

2	81.4% (79/97)	70.5% (62/88)	71.6% (68/95)	61.3% (57/93)	0.02
3	81.4% (79/97)	71.6% (63/88)	72.6% (69/95)	61.3% (57/93)	0.02
4	80.4% (78/97)	72.7% (64/88)	72.6% (69/95)	55.9% (52/93)	0.002
5	78.4% (76/97)	72.7% (64/88)	73.7% (70/95)	60.2% (56/93)	0.04
Pooled	80.4% (78/97)	71.6% (63/88)	72.6% (69/95)	60.2% (56/93)	0.02

Table S3. Multiple imputation results of HbA1c <7.0% at week 48

Datasets	LIN+MET	LIN	MET	Control	P
1	70.1% (68/97)	68.2% (60/88)	67.4% (64/95)	47.3% (44/93)	0.003
2	71.1% (69/97)	70.5% (62/88)	68.4% (65/95)	52.7% (49/93)	0.02
3	69.1% (67/97)	69.3% (61/88)	69.5% (66/95)	47.3% (44/93)	0.002
4	70.1% (68/97)	67.0% (59/88)	68.4% (65/95)	48.4% (45/93)	0.006
5	72.2% (70/97)	68.2% (60/88)	69.5% (66/95)	48.4% (45/93)	0.002
Pooled	70.1% (68/97)	68.2% (60/88)	68.4% (65/95)	48.4% (45/93)	0.005

Table S4. Multiple imputation results of HbA1c <6.5% at week 48

2.2 **Sensitivity analyses**

In these analyses, all participants in the full analysis set with missing 48-week HbA1c data were treated as not having achieved the primary endpoint of HbA1c <7%**.** The proportion of participants achieving HbA1c <7.0% at week 48 was 75.3% (73/97) group LIN+MET, 67.0% (59/88) in group LIN, 68.4% (65/95) in group MET, and 51.6% (48/93) in the control group (chi-square test overall P=0.003). After adjustment for multiple comparisons, the difference between the LIN+MET group and the control group reached statistical significance (P <0.001), whereas the LIN group vs. control group and MET group vs. control group did not (P=0.04 and P=0.02, respectively). Additionally, 63.9% (62/97), 59.1% (52/88), and 61.1% (58/95) of participants in the LIN+MET, LIN, and MET group achieved HbA1c <6.5%, compared with 37.6% (35/93) in the control group (chi-square test overall P <0.001; for LIN+MET vs control and MET vs control, P=0.001; for LIN vs control, P=0.004).

Logistic analysis, with trial centres set as a random effect, indicated that compared to the control group, the LIN+MET group (OR 3.28, 95% CI 1.51 to 5.29, P=0.02) and the MET group (OR 2.30, 95% CI 1.10 to 3.82, P=0.03) were more likely to achieve the HbA1c <7.0% endpoint,

while the relative effect in the LIN group was not significant (OR 1.70, 95% CI 0.92 to 3.06, P=0.09), after adjusting for age, gender, BMI, and baseline HbA1c levels. These results were generally consistent with those obtained from multiple imputation.

3. Safety issues of the study

	LIN+MET	LIN	MET	Control	P
No.	103	103	103	103	
Patients with any adverse event	46 (44.7%)	25 (24.3%)	42 (40.8%)	18 (17.5%)	<0.001
Related to the study medicines	20 (19.4%)	7 (6.8%)	18 (17.5%)	N.A.	0.02
Severe adverse events	1	0	0	1	-
Leading to drug discontinuation	3 (2.9%)	0	5 (4.9%)	N.A.	0.09
Death	0	0	0	0	-
All hypoglycemia events	5 (4.9%)	1 (1.0%)	3 (2.9%)	1 (1.0%)	0.21
Level 1 hypoglycemia	5 (4.9%)	1 (1.0%)	3 (2.9%)	1 (1.0%)	0.21
Level 2 or severe hypoglycemia	0	0	0	0	-
Gastrointestinal disorders	18 (17.5%)	7 (6.8%)	16 (15.5%)	4 (3.9%)	0.003
Nausea	11 (10.7%)	6 (5.8%)	9 (8.7%)	3 (2.9%)	0.14
Diarrhea	4 (3.9%)	1 (1.0%)	3 (2.9%)	0	0.17
Bloating	9 (8.7%)	3 (2.9%)	7 (6.8%)	2(1.9%)	0.09
Upper respiratory tract infection	8 (7.8%)	7 (6.8%)	10 (9.7%)	7 (6.8%)	0.85
Abnormal liver function	7 (6.8%)	5 (4.9%)	6 (5.8%)	3 (2.9%)	0.63
Rashes	6 (5.8%)	5 (4.9%)	7 (6.8%)	3 (2.9%)	0.63
Dizziness	3 (2.9%)	1 (1.0%)	2 (2.3%)	0	0.59
Hospitalization due to unplanned surgery	1 (1.0%)	0	0	1 (1.0%)	0.57
Cardiac symptoms	3 (2.9%)	3 (2.9%)	4 (3.9%)	3 (2.9%)	0.97
Urinary tract infection	2 (1.9%)	1 (1.0%)	2 (1.9%)	1 (1.0%)	0.88
Peripheral oedema	0	1 (1.0%)	1 (1.0%)	0	0.57
Hyperuricemia	2 (1.9%)	1 (1.0%)	3 (2.9%)	0	0.34

Table S5. Adverse events in the study population

4. Characteristics of participants who dropped out or were excluded from the efficacy analysis

Among the ITT set (n=412), 39 participants who did not return for any follow-up after SIIT were excluded from further efficacy analysis. Additionally, 52 participants were lost to follow-up after the week 12 visit. A total of 321 patients completed the 48-week follow-up (per-protocol set). Their baseline and post-SIIT parameters were comparable, with no significant differences (Table S6).

For those who dropped out, 31/52 (59.6%) did so after completing the 12-week follow-up, 19/52 (36.5%) after the 24-week follow-up, and 2/52 (3.8%) after the 36-week follow-up. Their baseline, post-SIIT, and last visit parameters are summarized in Table S7.

	Per-protocol participants	Participants who drop out after week 12	Participants Excluded from the efficacy analysis	P
No.	321	52	39	
Distribution				
Control	76	17	13	
LIN+MET	85	12	3	0.18
LIN	75	13	11	
MET	85	10	10	
Age (years)	47.1±10.7	45.2±12.2	46.7±13.3	0.53
Gender (F/M)	83/238	13/39	13/26	0.68
Estimated disease Duration (months)	1.0 (0.5,4.5)	2.0 (1,6.7.0)	1.0 (0.5,4.0)	0.06
SBP (mmHg)	130.4±16.5	124.7±14.2	131.2±16.2	0.06
DBP (mmHg)	83.3±11	80.8±9.6	82.4±12.2	0.29
BMI (kg/m^2)				
Before SIIT	25.7±2.7	26.1±3.7	26.0±3.2	0.56
After SIIT	25.2±2.7	25.9±3.7	25.5±3.1	0.32
Waist Circumference (cm)				
Before SIIT	91.8±8.3	92.1±10.0	90.9±8.4	0.83
After SIIT	90.5±8.0	91.9±9.9	92.2±8.5	0.36
Waist-to-hip ratio				

Before SIIT	0.9±0.1	0.9±0.1	0.9±0.1	0.94
After SIIT	0.9±0.1	0.9±0.1	0.9±0.1	0.36
Serum creatinine (μmol/L)	65.5±15.1	64.0±12.7	63.6±14.5	0.62
Alanine transaminase (U/L)	27.7±1.5	20.2±2.8	21.3±3.6	0.56
Cholesterol (mmol/L)				
Before SIIT	5.4±1.5	5.3±1.2	5.4±1.0	0.78
After SIIT	4.5±1.0	4.4±0.9	4.6±1.0	0.64
Triglyceride (mmol/L)				
Before SIIT	2.3±1.7	2.4±2.0	2.3±1.4	0.88
After SIIT	1.3±0.6	1.3±0.6	1.4±0.5	0.95
HDL-C (mmol/L)				
Before SIIT	1.1±0.5	0.9±0.2	1.0±0.2	0.37
After SIIT	1.1±0.3	1.0±0.2	1.1±0.3	0.17
LDL-C (mmol/L)				
Before SIIT	3.4±0.9	3.2±0.8	3.3±0.9	0.22
After SIIT	2.9±0.8	2.7±0.7	2.8±1.0	0.65
HbA1c (%)				
Before SIIT	10.9±1.9	11.1±1.7	11.3±1.6	0.48
After SIIT	9.2±1.3	9.3±1.2	9.4±1.2	0.46
FPG (mmol/L)				
Before SIIT	11.2±2.9	11.7±3.1	12.3±2.4	0.10
After SIIT	5.9±1.1	5.8±1.1	6.2±1.2	0.22
2hPG (mmol/L)				
Before SIIT	20.4±4.1	20.6±3.8	21.1±3.1	0.59
After SIIT	13.7±2.9	13.7±3.2	15.2±2.9	0.55
Matsuda index				
Before SIIT	84.1(59.4,122.2)	101.7(67.4,126.4)	78.4(63.5,122.8)	0.17

After SIIT	120.2(86.2,176.2)	141.4(83.7,191.4)	158.7(95.5,214.2)	0.22
ISSI-2				
Before SIIT	62.1(41.9,101.9)	58.8(43.4,85.9)	53.6(44,83.5)	0.58
After SIIT	264.9(200.9,369.2)	285(184.1,390.4)	214.5(151.6,302.7)	0.08
HOMA-IR				
Before SIIT	3.3(2.1,4.6)	2.8(2,3.8)	3(2.2,4)	0.19
After SIIT	1.3(0.8,2)	1.1(0.8,2)	1.1(0.7,2.3)	0.65
HOMA-β				
Before SIIT	17.7(10.6,31.2)	13(7.9,23.7)	12.5(8.2,22.8)	0.58
After SIIT	46.6(31.3,73.5)	44.2(27.3,77.8)	36.8(23.3,52.3)	0.29

Table S6. Characteristics of participants who were excluded from efficacy analysis, dropped out after week 12, or completed the trial (per-protocol). Data were presented as mean ± standard deviation for normally distributed data or as median (interquartile range) for non-normally distributed data. SBP, systolic blood pressure; DBP, diastolic blood pressure; BMI, body mass index; HDL-C, high-density lipoprotein- cholesterol; LDL-C, low-density lipoprotein-cholesterol; HbA1c, glycated hemoglobin A1c; FPG, fasting plasma glucose; 2hPG, 2h plasma glucose; ISSI-2, Insulin Secretion-Sensitivity Index-2; HOMA-IR, homeostasis model assessment of insulin resistance; HOMA-β, homeostasis model assessment of β cell function; SIIT, short-term intensive insulin therapy. The comparisons of parameters after SIIT have been adjusted for baseline values.

	Baseline	After SIIT	Last visit
Age (years)	45.2±12.2	-	-
Gender (F/M)	13/39	-	-
Estimated disease Duration (months)	2.0 (1.0, 6.7)	-	-
SBP (mmHg)	124.7±14.2	-	-
DBP (mmHg)	80.8±9.6	-	-
BMI (kg/m^2)	26.1±3.7	25.9±3.7	25.6±3.6
Waist Circumference (cm)	92.1±10.0	91.9±9.9	68.7±35.4
Serum creatinine (μmol/L)	64±12.7	-	-
Alanine transaminase (U/L)	20.2±2.8	-	-
Cholesterol (mmol/L)	5.3±1.2	4.4±0.9	5.0±1.0

Triglyceride (mmol/L)	2.4±2.0	1.3±0.6	2.2±2.4
HDL-C (mmol/L)	0.9±0.2	1.0±0.2	1.1±0.3
LDL-C (mmol/L)	3.2±0.8	2.7±0.7	3.0±0.8
HbA1c (%)	11.1±1.7	9.3±1.2	6.5±0.8
FPG (mmol/L)	11.7±3.1	5.8±1.1	7.0±1.7
2hPG (mmol/L)	20.6±3.8	13.7±3.2	10.8±5.3
Matsuda index	101.7 (67.4, 126.4)	141.4 (83.7, 191.4)	65.9 (39.1, 97.7)
ISSI-2	58.8 (43.4, 85.9)	285.0 (184.1, 390.4)	214.4 (80.7, 329.5)
HOMA-IR	2.8 (2.0, 3.8)	1.1 (0.8, 2.0)	3.1 (1.5, 5.0)
HOMA -β	13.0 (7.9, 23.7)	44.2 (27.3, 77.8)	43.3 (27.1, 86.8)
HbA1c < 7.0% at the last visit (n)*			
Control	12/17		
LIN + MET	6/12		
LIN	7/13		
MET	7/10		

Table S7. Characteristics of participants who dropped out of the trial after week 12. Data were presented as mean ± standard deviation for normally distributed data or as median (interquartile range) for non-normally distributed data. SBP, systolic blood pressure; DBP, diastolic blood pressure; BMI, Body mass index; HDL-C, high-density lipoprotein-cholesterol; LDL-C, low-density lipoprotein-cholesterol; HbA1c, glycated hemoglobin A1c; FPG, fasting plasma glucose; 2hPG, 2h plasma glucose; ISSI-2, Insulin Secretion-Sensitivity Index-2; HOMA-IR, homeostasis model assessment of insulin resistance; HOMA-β, homeostasis model assessment of β cell function; The comparisons of parameters after SIIT have been adjusted for baseline values; * Chi-square test P=0.60.

5. Detailed description of participants who required rescue anti-hyperglycaemic therapy during follow-up.

	Control	LIN	MET	LIN+MET	P
No.	15	10	9	7	N/A
Age (years)	46.7±11.5	53.6±15.9	47.5±12.5	41.7±10.7	0.31
Baseline BMI (kg/m2)	24.7±3.1	25.3±3.5	24.4±2.0	24.9±2.4	0.89
Baseline HbA1c (%)	10.1±1.8	12.2±1.0	11.4±1.3	11.5±1.9	0.04
Baseline FPG (mmol/L)	11.4±1.7	12.9±3.1	11.8±2.9	13.3±4.1	0.40
Baseline 2hPG (mmol/L)	19.8±2.7	22.3±2.3	21.3±4.6	20.3±4.2	0.42

Baseline Matsuda index	4.7(3.2, 7.6)	4.8(3.4, 7.3)	5.3(3.7, 6.7)	4.6(3.9, 9.7)	0.50
Baseline ISSI-2	61.6(51.0, 78.0)	65.5(33.1, 112.4)	52.8(36.4, 94.3)	44.5(38.1, 55.4)	0.09
Baseline HOMA-IR	2.8(1.8, 5.9)	3.2(2.1, 4.4)	2.8(2.3, 4.5)	3.1(1.5, 4.4)	0.71
Baseline HOMA -β	11.8(7.3, 35.1)	14(10.3, 17.7)	11.8(5.7, 26.7)	12(4.8, 20.2)	0.41
Time to Rescue event occured (weeks after SIIT)					
≤24 weeks	11	8	6	5	0.93
＞24 weeks	4	2	3	2	
HbA1c at rescue Therapy(%)	7.9±0.3	8.1±0.5	8±0.6	8.2±0.9	0.83
FPG at rescue therapy (mmol/L)	9.2±0.9	8.8±0.9	9.5±1.4	10.0±0.9	0.17
Type of rescue treatment					
Mono OHA	4	0	0	0	
Dual OHAs	6	9	7	0	
Triple OHAs	4	0	0	2	N/A
Basal insulin+OHA(s)	1	0	2	4	
GLP-1RA+OHA(s)	0	1	0	1	

Table S8. Characteristics of participants who required rescue anti-hyperglycaemic therapy during follow-up. Data were presented as mean ± standard deviation for normally distributed data or as median (interquartile range) for non-normally distributed data. HbA1c, glycated hemoglobin A1c; FPG, fasting plasma glucose; 2hPG, 2h plasma glucose; ISSI-2, Insulin Secretion-Sensitivity Index-2; HOMA-IR, homeostasis model assessment of insulin resistance; HOMA-β, homeostasis model assessment of β cell function; OHA, oral hypoglycaemic agent.

6. The impact of disease duration on primary outcome

In the FAS, 25.2% (94/373) of participants had an estimated disease duration of ≥6 months, with a maximum duration of 3 years, and there were no significant differences in disease duration across the treatment groups (control group: 25.8% [24/93], LIN+MET group: 28.9% [28/97], LIN group: 19.3% [17/88], MET group: 26.3% [25/95], chi-square test P=0.50). As shown in Table S9, the baseline clinical parameters for participants with different disease durations were comparable. We used the five datasets generated by multiple imputation of HbA1c data to calculate the primary endpoint for different

treatment groups. Sustained good glycaemic control can also be achieved in a large proportion of participants with a disease duration of ≥6 months. More participants with shorter durations may achieve the HbA1c endpoints, with a significant difference observed in the control group but not in the other three sequential treatment groups.

Of note, there is a limitation in estimating disease duration in this trial, as the exact onset time of hyperglycaemia in most T2DM cases cannot be precisely determined by self-reported clinical symptoms or the period of elevated blood glucose. Further evaluation is needed to assess the reproducibility and accuracy of the impact of estimated disease duration on clinical outcomes.

	≥6 months	＜6 months	
Age （years）	46.2±10.1	47.0±11.5	0.50
Duration (months)	1.0 (0.5, 1.0)	12.0(6.0, 24.0)	<0.001
BMI (kg/m^2)	25.5±2.6	25.9±3.0	0.57
HbA1c (%)	10.9±2.1	11.0±1.7	0.53
FPG (mmol/L)	11.9±2.7	11.1±3.0	0.05
2hPG (mmol/L)	20.6±3.6	20.4±4.2	0.68
HOMA -β	16.8(10.2, 31.2)	14.8(9.5,25.9)	0.22
HOMA-IR	3.0(2.0,4.4)	3.4(2.4,4.4)	0.23
Matsuda index	4.9(3.3,7.0)	4.6(3.4,5.8)	0.22
ISSI-2	63.7(42.9,102.0)	56.6(41.6,84.4)	0.14
Proportion of achieving HbA1c＜7.0 at week 48			
Control	45.8% (11/24)	73.9% (51/69)	0.02
LIN+MET	75.0% (21/28)	88.4% (61/69)	0.12
LIN	76.5% (13/17)	76.1% (54/71)	0.76
MET	72.0% (18/25)	78.6% (55/70)	0.58
Overall	67.0% (63/94)	79.2% (221/279)	0.03

Table S9. Baseline characteristics and primary outcome in participants with estimated disease duration of <6 months and ≥6 months. Data were presented as mean ± standard deviation for normally distributed data or as median (interquartile range) for non-normally distributed data. BMI, body mass

index; HbA1c, glycated hemoglobin A1c; FPG, fasting plasma glucose; 2hPG, 2-hour post-prandial plasma glucose; HOMA, Homeostasis model assessment; ISSI-2, Insulin Secretion-Sensitivity Index-2.
